# Medulla oblongata cavernoma removal through a lazy far lateral approach: operative video and technical nuances

**DOI:** 10.3171/2019.10.FocusVid.19430

**Published:** 2019-10-01

**Authors:** Carlos Candanedo, Samuel Moscovici, Sergey Spektor

**Affiliations:** Department of Neurosurgery, Hadassah Hebrew University Medical Center, Jerusalem, Israel

**Keywords:** brainstem, cavernoma, far lateral approach, medulla, video

## Abstract

Removal of brainstem cavernous malformation remains a surgical challenge. We present a case of a 63-year-old female who was diagnosed with a large cavernoma located in the medulla oblongata. The patient suffered three episodes of brainstem bleeding resulting in significant neurological deficits (hemiparesis, dysphagia, and dysarthria). It was decided to remove the cavernoma through a left-sided modified far lateral approach.^3^

The operative video demonstrates the surgical steps and nuances of a complete removal of this complex medulla oblongata cavernous malformation. Total resection was achieved without complications. Postoperative MRI revealed no signs of residual cavernoma with clinical improvement.

The video can be found here: https://youtu.be/BTtMvvLMOFM.

## Transcript

### 0:20 Introduction

This is Dr. Carlos Candanedo from Hadassah Hebrew University Medical Center in Jerusalem. I’ll be demonstrating a complex medulla oblongata cavernous malformation resection through a modified far lateral approach.

### 0:35 Patient clinical history

The patient is a 63-year old female who was diagnosed with a large cavernoma located in the medulla oblongata. After suffering three episodes of brainstem bleeding treated conservatively in another institution, she was referred for surgical evaluation.

### 0:53 Physical examination

On physical examination the patient was fully conscious, cooperative, and oriented. There was no deficit on cranial nerves but only on lower cranial nerves, having dysphagia and dysarthria. Besides, she had a mild left hemiparesis 4/5 and a significant right-side hemiparesis 3/5, but able to use the wheelchair.

### 1:19 Imaging studies

MRI revealed a 2-cm posthemorrhagic lesion in the medulla oblongata, with an exophytic part going out to the left anterolateral margin of the medulla, allowing for a surgical corridor to the lesion. There was no associated DVA (developmental venous anomaly).

### 1:37 Patient positioning

The surgery was performed in a right three-quarters prone position with neurophysiology monitoring including motor and somatosensory evoked potentials, facial nerve, ABR, and lower cranial nerves monitoring.

### 1:50 Approach

We used a previously described modified far lateral approach that we called lazy far lateral approach, through a lazy S retroauricular skin incision and performing a retrosigmoid suboccipital craniotomy, a C1 hemilaminectomy, and drilling of the posteromedial third of the occipital condyle to obtain good exposure ventral to the medulla.

We can appreciate the area of the retrosigmoid suboccipital craniectomy, with exposure of the foramen magnum, and the posterior arc of C1. Drilling of the posterior arc of C1. Validation of the vertebral artery with intraoperative Doppler. Partial mobilization of the vertebral artery with his periosteal sheath, in order to complete the hemilaminectomy as laterally as possible up to the transverse process of C1. You can see the vertebral artery liberated with his entrance on the atlantooccipital membrane.

The lateral edge of the foramen magnum rim is drilled away until the condylar fossa, including the posteromedial third of the condyle, to provide direct access to the anterior foramen magnum area.

### 3:14 Dura opening

The dura is opened in a C-shaped fashion, beginning as far lateral as possible near the sigmoid sinus, curving caudally and medially to the vertebral artery and beneath it obliquely as far lateral and inferior as possible, providing a dural flap.

In the left picture, we can see the resected left posterior arc of C1 and the suboccipital craniectomy. Once the dura is open, we continue with the arachnoid dissection.

### 3:47 Cavernous malformation exposure

We can see a typical multilobulated raspberry appearance of the cavernoma. Here there is the spinal accessory nerve. We tried to shrink the exophytic component while working between the vagus nerve. A difficult part was to dissect both vertebral arteries and vertebrobasilar junction while preserving anterior spinal arteries. We see how laterally we can be with this approach.

Now we can see the dissection on the part located dorsally to the vertebral arteries. You see the exit of the PICA. Now we separate the cavernoma from the anterior spinal artery of the opposite site. Last step of dissection from the exophytic part of this very fibrous cavernoma as a result of repeated hemorrhages.

Resection was done between this critical vessels; gliotic plane was seen without residual vessels suspicious of cavernoma.

### 4:55 Closure

Then hemostasis was verified and dural defect was sealed with a collagen matrix and cryothrombin mixture.

### 5:03 Postoperative MRI

Postoperative MRI showing a complete removal of this medulla oblongata cavernous malformation, without any neurological deterioration.

### 5:12 Postoperative course

The patient was discharged to a rehabilitation program, having a gradual improvement on her dysphagia and dysarthria. Two months after surgery the patient improved on her quadriparesis, and even was able to walk with a cane.
